# Quantifying maternal vaccination opportunities across gestational age windows: a real-world multi-country, longitudinal study

**DOI:** 10.1016/j.xagr.2025.100579

**Published:** 2025-10-19

**Authors:** Rachel Ford, Oluwatosin Nkereuwem, Ugochukwu Madubueze, Urudinachi Agbo, Suraj Bhattarai, Rabin Thami, Dan Kajungu, Victoria Nambasa, Agnes Msoka, Mir Mobarak Hossain, Edward P.K. Parker, Beate Kampmann

**Affiliations:** 1 Department of Clinical Research, London School of Hygiene & Tropical Medicine (Ford, Bhattarai, and Kampmann), London, UK; 2 Medical Research Council Unit, The Gambia at the London School of Hygiene and Tropical Medicine (Nkereuwem and Kampmann), Fajara, The Gambia; 3 National Obstetric Fistula Centre, Abakaliki, Nigeria (Madubueze); 4 Alex Ekwueme Federal University Teaching Hospital, Abakaliki, Nigeria (Madubueze and Agbo); 5 Global Health Research and Medical Interventions Institute, Nepal (Bhattarai and Thami); 6 Makerere University Centre for Health and Population Research, Kampala (Kajungu); 7 Department of Global Health, Stellenbosch University, Stellenbosch, South Africa (Kajungu); 8 African Union Development Agency, Johannesburg, South Africa (Nambasa); 9 Kilimanjaro Clinical Research Institute (Msoka), Moshi, Kilimanjaro, Tanzania; 10 Planning and Research Unit, Directorate General of Health Services, Dhaka, Bangladesh (Hossain); 11 Department for Infectious Disease Epidemiology and International Health, London School of Hygiene and Tropical Medicine (Parker), London, UK; 12 Centre for Global Health, Charité - Universitätsmedizin Berlin (Kampmann), Berlin, Germany

## Abstract

**Background:**

Maternal vaccination is a recognized strategy for reducing maternal and neonatal morbidity and mortality, including in low- and middle-income countries (LMICs). As new vaccines such as those for respiratory syncytial virus (RSV) and group B streptococcus (GBS) will be administered according to gestational age (GA), understanding real-world patterns of contact of pregnant women with antenatal care services (ANC) across pregnancy is essential to inform the most effective delivery strategies.

**Methods:**

We conducted a retrospective longitudinal, multisite analysis using anonymized data extracted from maternal health registers at ANC and delivery clinics in 6 LMICs: Bangladesh, The Gambia, Nepal, Nigeria, Tanzania, and Uganda. Eligible clinics contributed data from ANC and delivery services and access to routine data from January 2019 to December 2022. Retrospective, individual-level ANC attendance data from a total of 123,867 individuals were included in the study. These included the total number of ANC attendances per woman and GA at first and last ANC contact. The GA determination practices of each clinic were also recorded.

**Results:**

Across all sites, women had a median of 4 ANC contacts per pregnancy (IQR 2–5). An estimated 79·2% of women had at least one ANC contact during the 24–36-week GA window, falling to 66·6% in a 28−36 week GA window, and 46·5% at 32−36 weeks. When stratified by ANC contact frequency, the proportion with at least one contact in 24−36 weeks increased to 94·1% among women with ≥4 ANC contacts, indicating significantly greater vaccine delivery potential among those with more frequent ANC contacts.

**Conclusion:**

In these LMIC settings, the 24–36-week GA window presents opportunity for delivering maternal vaccination at high coverage. These findings underscore the need to align maternal vaccine delivery strategies with real-world ANC patterns to ensure equitable and timely vaccine access in LMICs.

**AJOG Global Reports at a Glance**

**Why was the study conducted?**

New vaccines to protect pregnant women and their babies from infectious diseases are currently being introduced and need to be given during specific gestational windows in pregnancy. We wanted to find out the best time to offer these vaccines in low-income settings.

**Key findings?**

Our data from over 120 000 pregnant women collected in 6 countries in Sub-Saharan Africa and South Asia show that a 24–36-week gestational age window would achieve highest coverage.

**What does this add to what is known?**

We show that attendance for antenatal care is much lower than the WHO recommended 8 visits, and if vaccines are only offered in the last trimester, significant numbers of women would miss out.

**Research in context**

**Evidence before this study**

We searched MEDLINE on July 16, 2025, for studies investigating the timing of antenatal clinic (ANC) contacts in low- and middle-income countries (LMICs), using the terms “((gestation* adj1 age*) OR timing OR timeliness OR timely) AND (antenatal OR ANC) AND (visit* OR attend* OR contact*))”, alongside a filter to restrict to studies in LMICs. Most studies on ANC attendance patterns focused on the total number of ANC contacts achieved rather than the gestational age (GA) at which those contacts occurred. Existing literature also explored the timing of the first ANC contact, determinants of maternal health service utilization, and ANC quality, but relatively few studies provided detailed insights into the distribution of ANC contacts across GA windows, particularly in relation to maternal immunization opportunities. A limited number of studies in Sub-Saharan Africa have assessed the timing and number of ANC contacts, including in relation to the World Health Organization’s recommendation for 8 or more contacts. With the exception of 2 studies from Kenya, the available research predominantly relies on publicly available data, rather than real-world data, and does not explore the potential for maternal vaccine delivery based on GA at ANC contact.

**Added value of this study**

This is the first multi-country, multi-site study using real-world ANC attendance data to understand vaccination opportunities across GA windows. With the recent licensure of a maternal vaccine against respiratory syncytial virus (RSV), and the likelihood of vaccination delivery through ANC systems, these findings provide timely and practical insights into ANC attendance patterns across 6 countries in Sub-Saharan Africa and South Asia. We found that nearly 80% of pregnant women had at least one ANC contact between 24 and 36 weeks’ gestation, indicating substantial potential for maternal vaccine delivery during this timeframe. Reachability declined significantly when shorter GA windows were considered. Among those who had 4 or more ANC contacts, reachability increased to over 94%, highlighting how improving the frequency of ANC contacts could considerably improve maternal immunization delivery.

**Implications of all the available evidence**

There are substantial opportunities for maternal vaccination at various GA windows across ANC clinics in Sub-Saharan Africa and South Asia. In particular, a 24–36-week GA window would offer potential for delivering maternal vaccination at high coverage.

## Introduction

Maternal vaccination is a proven and increasingly important strategy for reducing maternal and neonatal morbidity and mortality from infectious diseases.^1^^,^^2^ Vaccines administered during pregnancy, such as those for tetanus, pertussis, influenza, and COVID-19 have demonstrated safety and effectiveness.^3^, ^4^, ^5^, ^6^, ^7^ A vaccine against respiratory syncytial virus (RSV) is now licensed, and new vaccines targeting group B streptococcus (GBS) are in the clinical development pipeline.^8^, ^9^, ^10^ Successful implementation of maternal vaccines in low- and middle-income countries (LMICs) will depend not only on vaccine acceptability and supply,^11^ but also on the capacity of antenatal care (ANC) systems to deliver them at the appropriate gestational age (GA).^11^^,^^12^

Timing is critical to the effectiveness of maternal immunisation.^3^ To optimize immune protection for both mother and infant, vaccines are typically recommended within specific GA windows.^13^ For example, the licensed RSV vaccine is currently recommended between 24 and 36 weeks’ gestation in over 40 countries.^14^ However, some regulatory authorities recommend narrower administration windows,^3^ which may pose significant challenges in LMICs, where late presentation to ANC, inconsistent gestational dating, and health system limitations are common.^11^^,^^12^

Despite global licensure, the maternal RSV vaccine has yet to be approved in sub-Saharan Africa or any LMICs, other than India, despite more than 90% of RSV-related deaths occurring in low- or lower-middle-income countries.^11^ Understanding when and how pregnant women access ANC is therefore essential to inform maternal vaccine implementation strategies and ensure equitable access in the settings that bear the greatest burden of disease.

While there is some understanding of GA at the first ANC presentation,^15^, ^16^, ^17^, ^18^, ^19^, ^20^ real-world data on the frequency and timing of ANC contacts across different GA windows in LMICs remain limited.^21^^,^^22^ This evidence gap hinders accurate estimation of achievable vaccine reachability. With the recent licensure of an RSV vaccine, and continued progress toward a maternal GBS vaccine, there is an urgent need to understand how to best reach pregnant women through ANC settings during specific GA windows.

In this study, we analyzed real-world ANC attendance patterns across multiple LMIC settings, with a particular focus on the frequency and timing of contacts in order to identify the opportunities for greatest coverage.

## Methods

### Study setting

We conducted a multi-country, multi-site, retrospective longitudinal data extraction study spanning January 2019 to December 2022. The study protocol has been previously published.^23^

Anonymized data were extracted from maternal registries held at ANC and delivery clinics in Bangladesh, The Gambia, Nepal, Nigeria, Tanzania, and Uganda. Local Co-Investigators identified 4 to 5 ANC clinics in their respective countries. Clinics were eligible for inclusion if they provided both ANC and delivery services; had an average of 50 births and/or 50 ANC contacts per month; and had a minimum of 4 years of ANC documentation (January 2019 to December 2022). Gaps in data collection due to the COVID-19 pandemic were accepted. Clinics were excluded if they specialized in complicated pregnancies only or if they were predominantly referral-based. To ensure representation of different healthcare settings and delivery patterns, country investigators included 2 urban and 2 rural clinics, with the potential to include one additional private clinic. Clinics were assigned an identifying code to support subgroup analyses while avoiding direct disclosure of information relating to specific facilities.

### Data extraction

Data were collected over a 4-year period covering January 2019 to December 2022, allowing analysis of potential impacts of the COVID-19 pandemic on ANC attendance patterns and documentation. A co-developed data extraction form (see appendix) was used to collect retrospective individual-level ANC attendance data, and each clinic’s GA determination practices.

Routine methods used to determine GA at each clinic were documented. These included fundal height measurement, recall of the last menstrual period (LMP), and ultrasound assessment. Clinics could report the use of one or more methods, with the reporting of a specific method indicating that it was used at least occasionally in routine practice.

Individual-level data were extracted from all relevant maternal registries held at ANC and delivery clinics. Specifically, antenatal, ⁠post-natal, and delivery registers, as well as any additional registries or notebooks found at clinics. Extracted data were entered into a country-specific REDCap database using electronic data collection tools (tablets, smartphones).^23^

Data collectors recorded individual-level data, including the total number of ANC attendances per woman, and GA at first and last ANC contact. In Nepal, data were collected using the same methodology, however, 2 participating clinics (NP1 and NP4) did not routinely document ANC contacts prior to the fourth contact. As a result, data for women with fewer than 4 recorded contacts at these clinics were unavailable and could not be included in the analysis. Total attendances per woman were obtained to understand the number of potential opportunities to administer a vaccine, while GA at first and last contact allowed us to map the timing of ANC attendances across a pregnancy.

Due to logistical and workforce constraints, it was not realistic to collect individual-level attendance data on every woman attending ANC for the duration of the 4-year period. Therefore, upon agreement across all study clinics, individual-level data were collected from all available records of women who attended the clinics during the first week of every month. This data collection method ensured that our data reflected attendances across seasons and years while still providing a large and representative sample size from each participating clinic.

### Data management

Individual country databases and REDCap quality control were managed by country-level data managers. The REDCap databases were designed to minimise errors and included parameters that simplified the formal data verification and cleaning process. Throughout data entry and cleaning, individual country REDCap databases were monitored by the country Co-Investigators and the Data Management team in the MRC Unit The Gambia.^23^

All databases were located on a secure network with restricted, password-protected access. Individual-level data were assigned a unique identifier at sites, unique to each country, clinic, and timeframe. No personal identifiers were collected.

### Data analysis

We performed an initial descriptive analysis of the total number of ANC contacts by country alongside GA at first and last contact. Individuals were excluded from this analysis if: the number of ANC contacts was not recorded; a total of one ANC contact was recorded but separate GAs were entered under first and last contact; or the ANC clinic was not recorded. Individuals with >16 ANC contacts were excluded from the database given that they represent outliers that are not representative of typical health-seeking patterns. Combined metrics across countries were generated by (1) summing country-level data; and (2) normalising country-level data via inverse probability weighting (i.e., assigning less weight to individuals from countries contributing larger cohorts). We performed secondary analyses stratified by ANC clinic and year. For the analysis of GA at first and last contact, we performed analyses overall and stratified by number of ANC contacts (1, 2–3, and ≥4).

Individual-level data were then used to estimate the number of women that could be reached for vaccination in a range of GA windows of interest, including 0–23 weeks, 24–36 weeks, 28–36 weeks, and 32–36 weeks. These windows were selected to reflect current regulatory guidance and anticipated future recommendations for the timing of maternal vaccine administration (FDA,^24^ EMA,^25^ UKHSA,^26^ WHO^14^).

Individuals were excluded from the analysis of GA windows if: the GA was missing for either first or last contact; or the GA at last contact was lower than the GA recorded at first contact (i.e., reflecting a data extraction error).

As GA data were only collected on first and last ANC contact, we interpolated between the first and last contacts under a range of assumptions to estimate attendance prevalence in GA windows. Our main analysis approach involved random interpolation of unobserved contacts between the first and last (allowing multiple contacts per week). As sensitivity analyses, we assessed: (1) even interpolation between first and last ANC contact; and (2) no interpolation (limited to observed contacts; see Supplementary Table S1 for summary and illustrative example). We performed secondary analyses stratified by: ANC clinic, year, and number of ANC contacts (1–3 vs and ≥4).

## Results

After data filtering, a total of 123,867 individuals were included in the study (Supplementary Table S2), including 33,950 from Bangladesh, 20,918 from The Gambia, 9645 from Nepal, 41,065 from Nigeria, 6,332 from Tanzania, and 11,957 from Uganda.

During data extraction, data collectors identified several clinics with incomplete records for limited time periods. These gaps were primarily due to temporary clinic closures, most commonly related to renovations, the COVID-19 pandemic, or the unavailability of ANC registers, which had been lost, damaged, or removed during infrastructure changes or local disruptions. For each instance, data collectors documented the affected clinics in the affected timeframe and the underlying reason (Supplementary Table S3).

**Figure 1:**
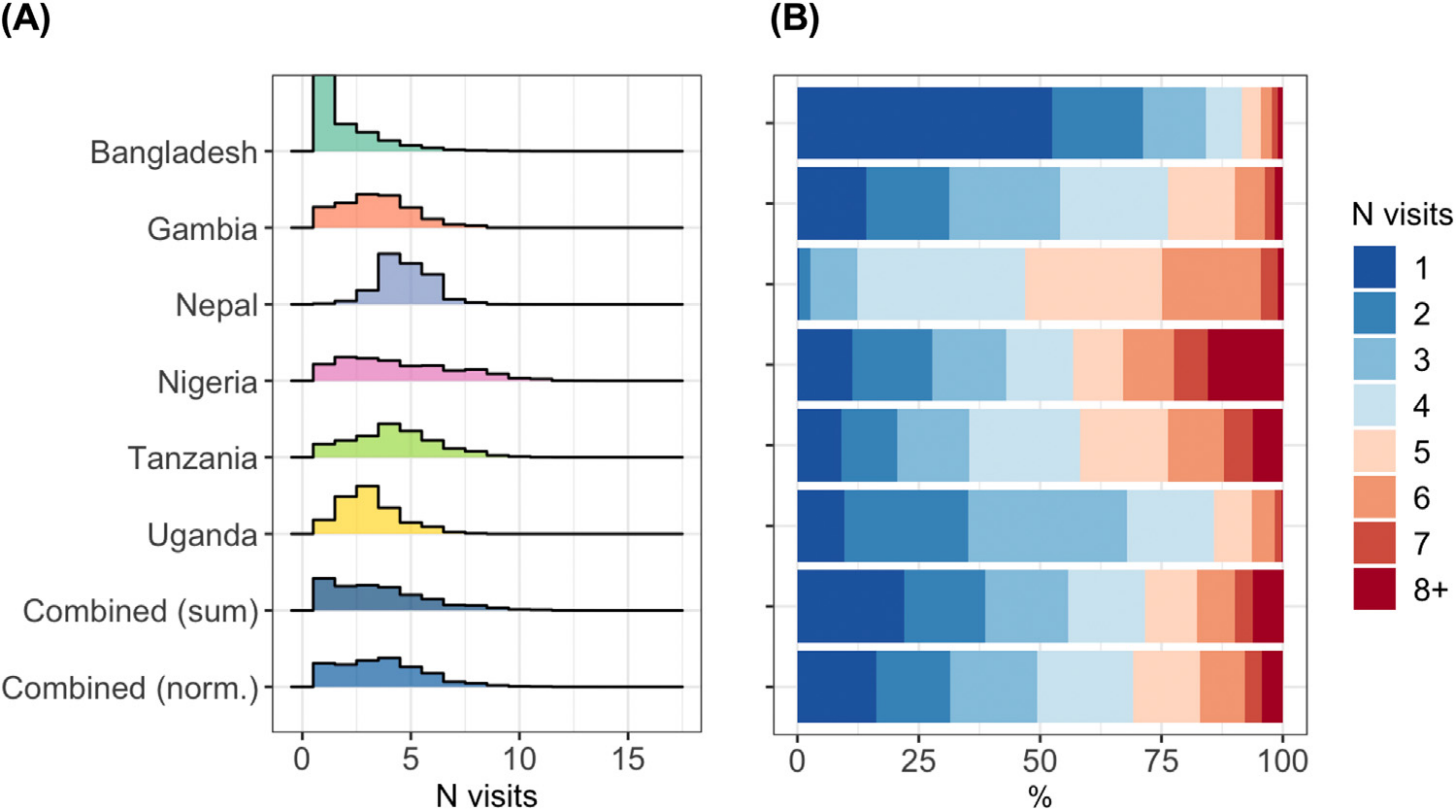
Distribution in the number of antenatal clinic contacts by country **(A)** Density plot of number of antenatal clinic contacts by country. **(B)** Stacked bar chart of antenatal clinic contact count by country. See Supplementary Table S3 for underlying data. Norm., Normalized by inverse probability weighted scaling at country level. *Ford: Quantifying maternal vaccination opportunities across gestational age windows: a real-world multi-country, longitudinal study. AJOG Glob Rep 2025.*

Based on normalized estimates combined across the 6 countries, we documented a median of 4 (interquartile range [IQR] 2–5) ANC contacts across pregnancies (Figure 1 and Supplementary Table S4). This included 16·2% of women attending ANC only once during their pregnancy, 15·2% attending ANC twice, 18·0% attending 3 times, and 50·7% attending 4 or more times.

There was significant variation in the number of ANC contacts per pregnancy by country (Figure 1 and Supplementary Table S4), with median counts per pregnancy ranging from 1 (IQR 1–3) in Bangladesh to 5 (IQR 4–6) in Nepal. Bangladesh was distinct in that the majority of women (52·4%) attended ANC clinics only once during pregnancy, whereas in other countries this proportion ranged from 0·4% (in Nepal) to 14·2% (in The Gambia).

The proportion of women attending ANC clinics ≥4 times ranged from 15·8% in Bangladesh to 87·8% in Nepal (Supplementary Table S4). The proportion of women attending ANC clinics ≥8 times ranged from 1·1% in Bangladesh to 15·6% in Nepal (Supplementary Table S4).

The distribution of ANC contact counts was broadly consistent across clinics in a given country, albeit with some deviations (Supplementary Figure S1 and Supplementary Table S5). For example, in Bangladesh, the proportion of individuals with one recorded contact was higher in clinics BD2 and BD5 than elsewhere. Although ANC contact counts were also consistent from year to year, a modest uptick in 2021 and 2022 was evident in Bangladesh (likely driven in part by a rise in contributions from BD1; Supplementary Table S3), The Gambia, and Tanzania (Supplementary Figure S2 and Supplementary Table S6).

**Figure 2:**
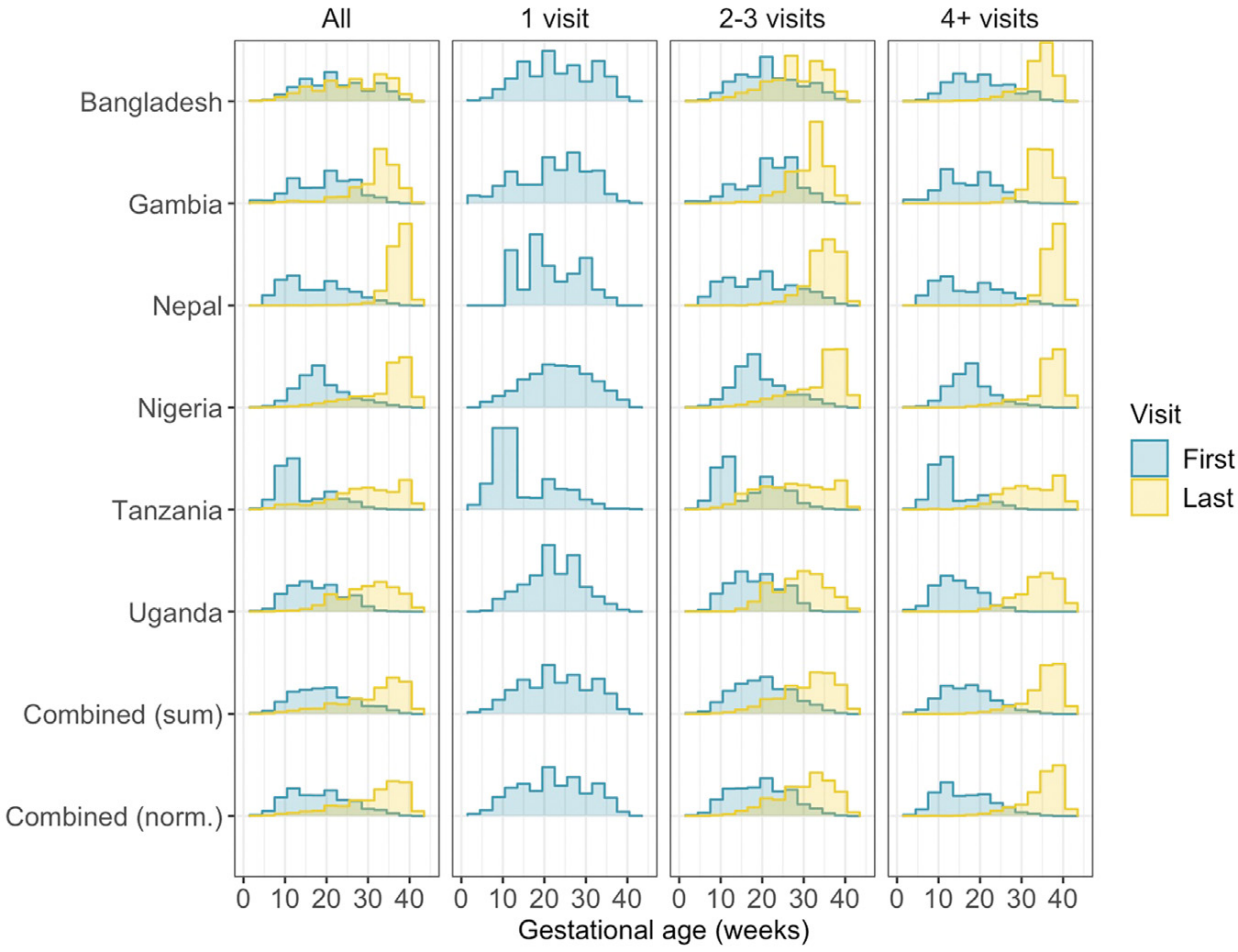
Distribution of gestational age at first and last antenatal clinic contacts by country Density plots are shown overall and stratified by total ANC contact count (1, 2–3, 4+). See Supplementary Table S4 for underlying data. Norm., Normalized by inverse probability weighted scaling at country level. *Ford: Quantifying maternal vaccination opportunities across gestational age windows: a real-world multi-country, longitudinal study. AJOG Glob Rep 2025.*

Overall, the median GA at first contact was 18 weeks (IQR 12–24) and the median GA at last contact was 34 weeks (IQR 27–37). First ANC contacts tended to occur earlier in Tanzania (median 11; IQR 10–19) than in other countries (Figure 2 and Supplementary Table S7). Median GA at last contact varied from 27 (IQR 20–34) in Bangladesh to 38 (IQR 36–39) in Nepal.

Across all countries, women with more ANC attendances had a lower GA at first contact and a higher GA at last contact than women with less ANC attendances. For example, for women who attended ANC 2–3 times, the median GA at first contact was 20 weeks (IQR 14–24) and the median GA at last contact was 31 weeks GA (IQR 26–36) (Supplementary Table S7). By contrast, women who attended 4 or more times first presented to ANC at a median GA of 16 weeks (IQR 11–21) and last presented at a median GA of 36 weeks (IQR 33–38).

The distribution of GAs at first and last ANC contact was consistent across clinics in a given country (Supplementary Figure S3 and Supplementary Table S8) and from across years (Supplementary Figure S4).

Among the 28 participating clinics, 96·4% (27 clinics) reported using more than one method to determine GA. All clinics (100%) used fundal height measurements, 85·6% relied on recall of the LMP, and 82·1% (23 clinics) reported some use of ultrasound. However, the reported use of ultrasound did not reflect systematic or regular use (Supplementary Table S3).

When randomly interpolating the GA of unobserved ANC contacts between first and last, an estimated 79·2% of women attended at least one ANC contact during the 24–36-week GA window, representing the largest opportunity for vaccination with country-specific estimates of 60·2% in Bangladesh, 89·7% in The Gambia, 98·7% in Nepal, 82·3% in Nigeria, 75·2% in Tanzania, and 81·3% in Uganda. When the window was narrowed to 28–36 weeks, the proportion of women with at least one contact decreased to 66·6%, with country-specific estimates ranging from 46·5% in Bangladesh to 86·0% in Nepal. A further restriction of the window to 32–36 weeks resulted in a marked decline in opportunity for vaccination, with only 46·5% of women attending at least once during this period, with country-specific estimates ranging from 31·2% in Bangladesh to 69·5% in Nepal.

**Figure 3:**
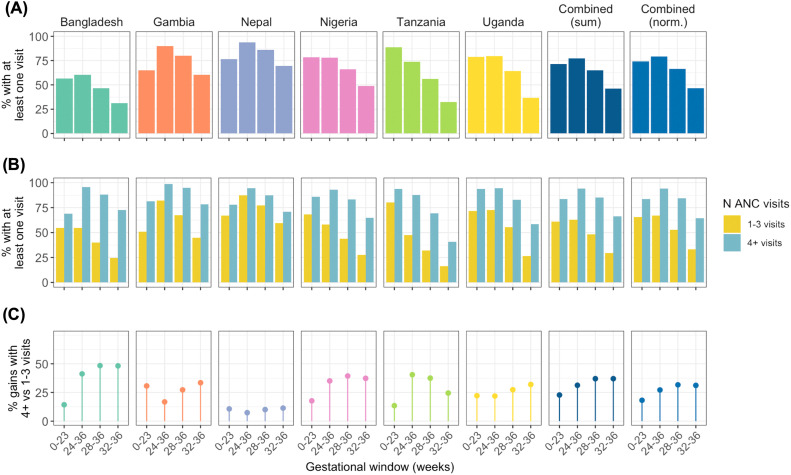
Proportion of individuals with at least one antenatal clinic contact in specific gestational age windows Unobserved ANC clinic contacts were randomly interpreted between the first and last contacts. Results are shown **(A)** overall and **(B)** stratified by total ANC contact count (1–3, 4+). **(C)** Percentage gain in contacts in specific windows according to total ANC contact count. See Supplementary Tables S9 and S12 for underlying data. Norm., Normalized by taking mean of country-specific estimates. *Ford: Quantifying maternal vaccination opportunities across gestational age windows: a real-world multi-country, longitudinal study. AJOG Glob Rep 2025.*

Attendances earlier in gestation present an opportunity for administration of other maternal vaccines where recommended (e.g. tetanus). Overall, 74·0% of women attended ANC clinics at least once during the 0–23-week GA window, with country-specific estimates ranging from 56·5% in Bangladesh to 88·8% in Tanzania (Figure 3).

Estimated attendances in each GA window of interest were generally consistent from clinic to clinic, albeit with some variation across clinics in Bangladesh reflecting the variation in attendance patterns described above. There was limited variation in estimated attendance patterns from year to year.

When stratified by ANC contact frequency, attendances in each GA window were consistently higher in women with ≥4 vs 1–3 contacts (Figure 3B). In the 24–36-week window, attendances increased from 66·9% to 94·1% (a 27·2% increase) in women attending 1–3 vs ≥4 times based on normalised estimates across countries (Figure 3C), with country-specific increases ranging from 7·4% in Nepal to 41·2% in Bangladesh. Similarly, there was an overall increase by 31·7% in contacts at 28–36 weeks (from 52·6% to 84·3% in women with 1–3 vs ≥4 contacts) and by 31·2% in contacts at 32–36 weeks (from 33·1% to 64·3% in women with 1–3 vs ≥4 contacts). Among women with ≥4 contacts, the percentage attending at least once in the 24–36-week window ranged from 87·1% in Tanzania to 98·7% in The Gambia.

Findings were highly consistent in sensitivity analyses that applied even as opposed to random interpolation of unrecorded contacts. Without interpolation (i.e., when limiting to GA at first and last contacts), representing a highly conservative estimate of vaccination opportunities, the estimated attendances in specific windows dropped (e.g., from 79·2% to 61·1% during the 24–36-week window).

## Discussion

With the introduction of novel vaccines that are specifically designed for use in pregnancy but recommended to be administered during specific GA windows (e.g., RSV), an understanding of the associated practicalities of administration have become ever more pressing. In light of real-world data, policy recommendations now require an assessment to successfully guide implementation strategies.

This study set out to provide an in-depth analysis of real-world ANC attendance patterns, with a particular focus on opportunities to vaccinate women in line with specific GA windows. Despite its setting in 6 different low-income countries and 2 continents, a relatively consistent picture emerged.

Our findings show that nearly 80% of pregnant women attend an ANC clinic at least once between 24 and 36 weeks’ gestation, offering considerable potential for maternal vaccine delivery in this window. Similarly, 2 recent studies in Kenya found between 85% and 96% of women attended ANC at least once during this window.^27^^,^^28^ We found that the potential reachability would decline to 66·6% if a vaccine was offered between 28 and 36 weeks, and drop to just 46·5% for a 32–36 week window. Notably, among women who attended 4 or more ANC contacts, reachability rose sharply to 94·1%, highlighting how increasing ANC contact frequency could be a highly effective strategy to optimize maternal vaccine delivery., This finding is supported by Nyiro et al.,^27^ who reported that women with a greater number of ANC contacts (up to 5 recorded) were more likely to have visits occur within key GA windows.

Our real-world evidence on ANC attendance patterns across diverse settings, highlighted the gap between global targets and current practice. Despite a median of 4 ANC contacts across the cohort, there was substantial variation between countries. For example, women in Bangladesh had a median of just 1 contact while those in Nepal reached a median of 5 contacts, short of the World Health Organization (WHO)-recommended 8 contacts per pregnancy.^29^

In 2016, WHO recommendations changed from a minimum of 4 to 8 ANC contacts per pregnancy in order to enhance maternal and neonatal outcomes and improve the overall pregnancy experience.^29^ However, compliance remains limited in many resource-constrained settings.^16^^,^^30^

While there was no significant variation in the number of ANC contacts between years, country-level variation was notable. This variation is consistent with the UNICEF global databases.^31^ We found fewer than 2% of women in Bangladesh, The Gambia, Nepal, and Uganda met the recommended threshold of 8 ANC contacts, compared to 6·0% in Tanzania and 15·6% in Nigeria. These disparities may partly reflect differences in health systems and sociocultural barriers, but also highlight inconsistencies in data recording practices. For instance, Nepal’s routine antenatal documentation is only retained for women who attend 4 or more ANC contacts for practical reasons. This introduces a positive bias in estimates of ANC contact counts and vaccination opportunities in this country (given the under-representation of individuals with <4 ANC contacts).

Our findings of low ANC contact counts per pregnancy are consistent with other reports ^16^^,^^18^^,^^22^^,^^30^^,^^32^ though the proportion of women receiving 8 or more ANC contacts was lower than that reported in several previous studies. An analysis of data from 54 countries, including all countries in our study except Bangladesh, reported that just 11·3% of women attended 8 or more ANC contacts, while 60·6% reached at least 4 contacts.^16^ Similarly, 3 analyses from sub-Saharan Africa found only 9%–10% of women achieved 8 or more ANC contacts.^18^^,^^22^^,^^30^ One study from Eastern Uganda found 23·4% of women reached 8 or more contacts, much higher than in our study population.^33^ One analysis of Nigerian DHS data reported 18·9% of women reaching 8 or more ANC contacts, which falls in a similar range to our findings.^18^

Following its recent approval, the WHO recommends integrating maternal RSV vaccination into routine ANC contacts in alignment with its core package of care,^14^ which recommends 5 of 8 ANC contacts occurring during the third trimester.^29^ WHO also recommended more implementation research, given that the success of this strategy will depend on whether pregnant women are in fact accessing ANC services during those gestational windows.

The discussion surrounding the GA window of RSV vaccine delivery was prompted by a potential association between RSV vaccination with preterm birth in clinical trials,^34^^,^^35^ though there is ongoing debate over the interpretation of these findings and subsequent data following implementation in several countries are reassuring.^36^

Although no causal relationship between maternal RSV vaccination and preterm birth has been established,^34^ many regulatory authorities have adopted a precautionary approach, recommending administration only in the third trimester to minimize potential risks of premature delivery.^3^^,^^11^^,^^14^ These gestational limits have contributed to considerable variation in global regulatory guidance. For instance, the European Medicines Agency,^25^ the UK Joint Committee on Vaccination and Immunisation,^26^ and Australia’s Therapeutic Goods Administration^37^ have authorized administration from 24 to 36 weeks. In contrast, WHO recommends vaccination during the third trimester, defined in accordance with national guidelines (generally ≥28 weeks),^14^ while the US Food and Drug Administration^24^ and the Public Health Agency of Canada^38^ have approved the vaccine for use between 32 and 36 weeks’ gestation.

The resulting heterogeneity in policy recommendations has significant implications for programmatic delivery. As our data show, restricting vaccination to narrower GA intervals, such as 32–36 weeks, may limit vaccine coverage substantially, particularly in settings where ANC attendance is less frequent or less timely. Delaying vaccination to the third trimester risks missing protection against RSV for the most vulnerable newborns, if indeed the pregnancy ends before term.

Consistent with previous findings, we found that nearly all participating clinics reported using multiple approaches to determine GA. All clinics reported routine use of fundal height, and while ultrasound was reportedly available at most clinics, it was not used systematically and LMP and fundal height remained the predominant methods in routine practice. This continued reliance on less precise methods raises concerns about the accuracy of GA estimates in ANC settings, including in the context of maternal vaccination.

Accurate GA dating is also important for establishing reliable preterm birth rates, which vary by setting and are key for interpreting vaccine study outcomes.

Our study possesses several key strengths that enhance its validity and relevance: first, it addresses a critical evidence gap by providing real-world data on ANC attendance by GA across multiple clinics in 6 low-income countries. The data reflect routine service delivery rather than conditions influenced by heightened observation or study interventions, thereby improving generalizability to typical care settings. Second, data collection spanned a 4-year period, capturing temporal trends and enabling assessment of the impact of the COVID-19 pandemic on ANC attendance.

This study also has several limitations. The data may not be nationally representative as they reflect only a subset of women attending ANC services in selected regions of each country. Clinic selection was limited to 4 or 5 locations per country, with a mix of facilities, potentially influencing generalizability of the findings. Due to the large dataset, data were only collected from ANC contacts occurring during the first week of each month. Additionally, women who transferred between facilities during pregnancy may not have been captured.

Data collection for this study was logistically complex and resource-intensive due to the absence of electronic health records at most clinics. Gestational age at ANC contacts and contact frequency had to be manually extracted from paper-based registries, introducing potential for data loss and transcription errors. The implementation of electronic registries could streamline data collection and enable linkage with pregnancy outcomes, allowing for more robust surveillance.^39^ Such systems are particularly critical in the context of maternal immunization, where accurate and timely data on adverse events of special interest (AESI) are essential for monitoring vaccine safety.

In conclusion, our study highlights substantial opportunities for maternal vaccination across various gestational age windows. In general, the 24–36-week GA window presents opportunity for delivering maternal vaccination at high coverage. Safe and effective implementation of novel maternal vaccines would be facilitated by reliable GA dating and the availability of digitalized medical record systems in low-resource settings to enable more reliable data collection.

## Patient consent statement

Routinely collected and fully anonymized data were extracted from maternal registries held at ANC and delivery clinics in Bangladesh, The Gambia, Nepal, Nigeria, Tanzania, and Uganda. No personal identifiers were collected. Individual patient consent was therefore not required as per Ethical committee decisions across all study sites.

## Data availability

Anonymized data and analytic code are available on Github at https://github.com/eparker12/GRGR_antenatal_care_attendance.

De-Identified individual participant data (text, tables, figures, and appendices) are available indefinitely, as well as the analytic code, at https://github.com/eparker12/GRGR_antenatal_care_attendance. This data is available immediately to anyone wishing to access this data, and for any purpose. The study protocol is already available.^23^

## CRediT authorship contribution statement

**Rachel Ford:** Writing – original draft, Validation, Supervision, Project administration, Methodology, Formal analysis, Data curation. **Oluwatosin Nkereuwem:** Writing – review & editing, Supervision, Project administration, Investigation, Data curation. **Ugochukwu Madubueze:** Writing – review & editing, Validation, Methodology, Investigation, Data curation. **Urudinachi Agbo:** Writing – review & editing, Supervision, Project administration, Methodology, Investigation, Data curation, Conceptualization. **Suraj Bhattarai:** Writing – review & editing, Validation, Project administration, Investigation, Formal analysis, Data curation, Conceptualization. **Rabin Thami:** Writing – review & editing, Validation, Project administration, Data curation. **Dan Kajungu:** Writing – review & editing, Validation, Project administration, Methodology, Investigation, Data curation. **Victoria Nambasa:** Writing – review & editing, Validation, Methodology, Investigation. **Agnes Msoka:** Writing – review & editing, Supervision, Project administration, Methodology, Investigation, Data curation. **Mir Mobarak Hossain:** Writing – review & editing, Validation, Project administration, Methodology, Investigation, Data curation. **Edward P.K. Parker:** Writing – review & editing, Visualization, Methodology, Investigation, Formal analysis, Data curation. **Beate Kampmann:** Writing – original draft, Validation, Supervision, Resources, Project administration, Methodology, Investigation, Funding acquisition, Formal analysis, Conceptualization.

## Appendix Supplementary materials

Image, application 1

## Footnotes

Supplementary material associated with this article can be found in the online version at doi:10.1016/j.xagr.2025.100579.
