# Supplementary material for: Quantifying maternal vaccination opportunities across gestational age windows: a real-world multi-country, longitudinal study
Source: AJOG Glob Rep. 2025 Oct 19;5(4):100579. doi: 10.1016/j.xagr.2025.100579 (PMC12670892; doi:10.1016/j.xagr.2025.100579)
Supplement: Supplementary file 1 [file mmc1.docx]

**Supplementary Appendix**

**Quantifying Maternal Vaccination Opportunities across Gestational Age Windows: a Multi-country, Longitudinal Study of Antenatal Care Attendance**

**Ford, et al**

**Contents**

P2 Supplementary Figure S1. Distribution in the number of antenatal clinic visits by clinic.

P3 Supplementary Figure S2. Distribution in the number of antenatal clinic visits by year.

P4 Supplementary Figure S3. Distribution of gestational age at first and last antenatal clinic visits by clinic.

P5 Supplementary Figure S4. Distribution of gestational age at first and last antenatal clinic visits by year.

P6 Supplementary Table S1. Interpolation approaches for unobserved visits.

P7 Supplementary Table S2. Selection of individual-level data for inclusion in the study.

P8 Supplementary Table S3. Distribution of antenatal clinic visit counts by country.

P9 Supplementary Table S4. Distribution of antenatal clinic visit counts by clinic.

P10 Supplementary Table S5. Distribution of antenatal clinic visit counts by year.

P11 Supplementary Table S6. Distribution of gestational age at first and last antenatal clinic visit by country.

P12 Supplementary Table S7. Distribution of gestational age at first and last antenatal clinic visit by clinic.

P13 Supplementary Table S8. Distribution of gestational age at first and last antenatal clinic visit by year.

P14 Supplementary Table S13. Proportion of individuals with at least one antenatal clinic visit in specific gestational age windows by total antenatal clinic visit count.

**(A (B)**


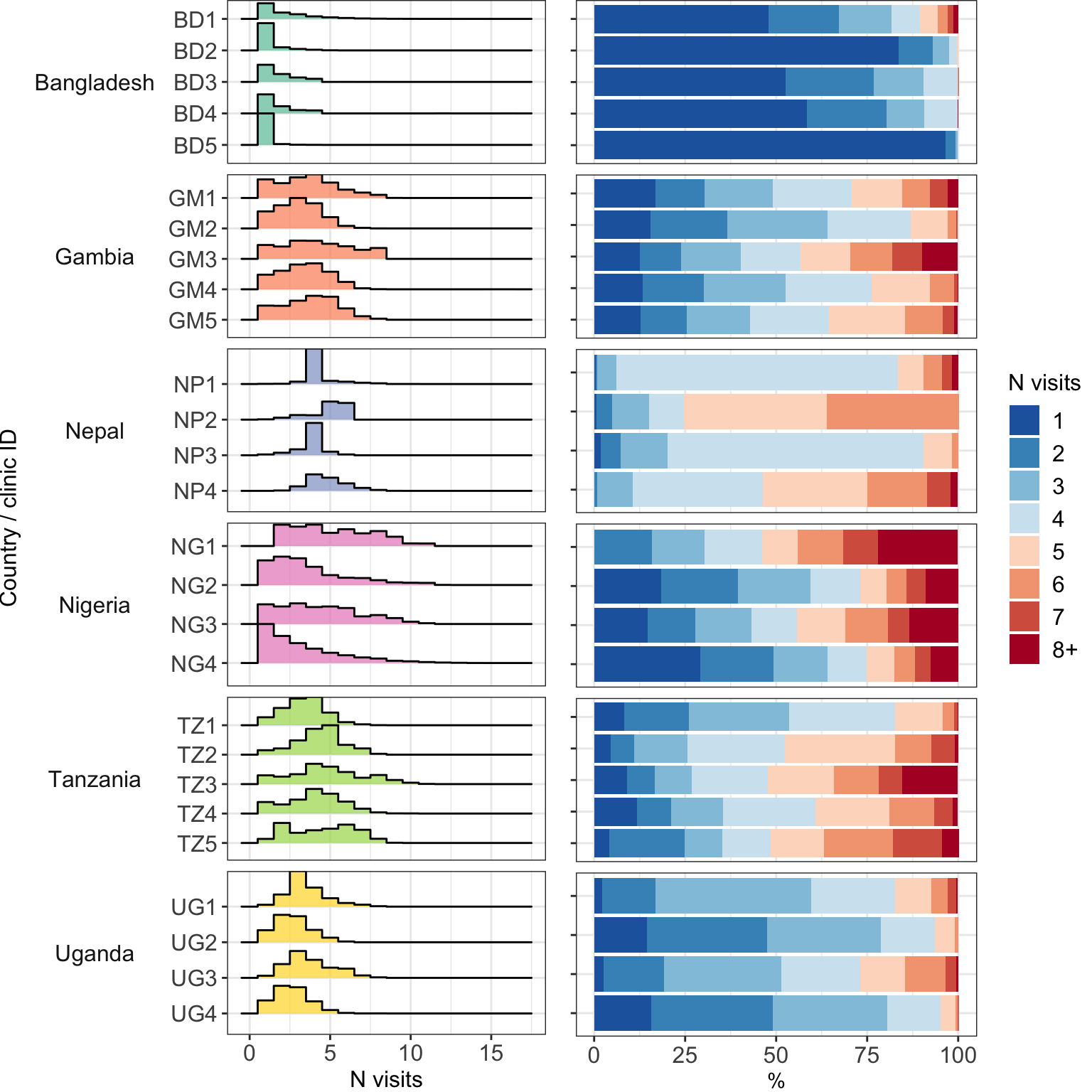


**Supplementary Figure S1. Distribution in the number of antenatal clinic visits by clinic. (A)** Density plot of number of antenatal clinic visits by clinic. **(B)** Stacked bar chart of antenatal clinic visit count by clinic. See **Supplementary Table S4** for underlying data.

**(A) (B)**

**
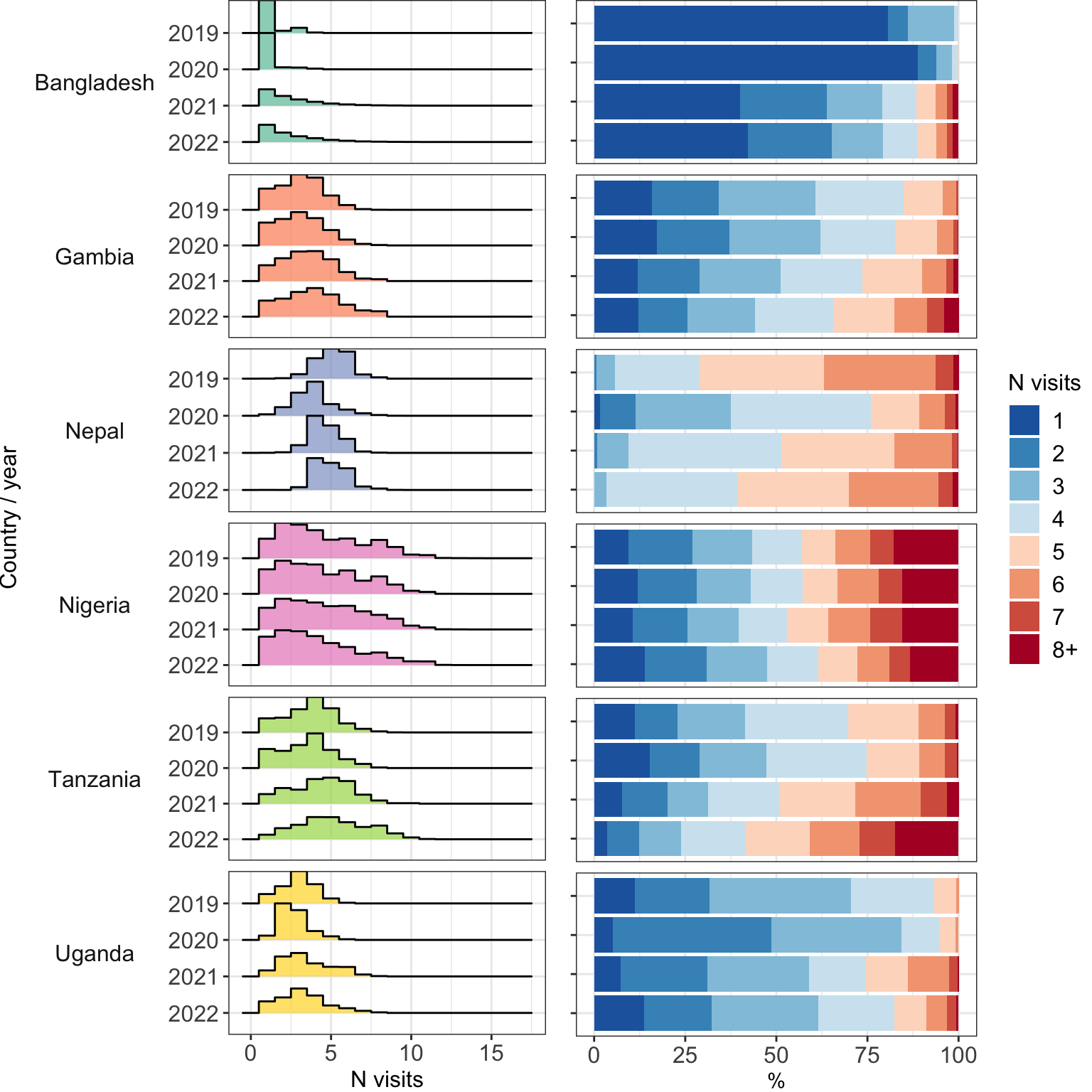
**

**Supplementary Figure S2. Distribution in the number of antenatal clinic visits by year. (A)** Density plot of number of antenatal clinic visits by year. **(B)** Stacked bar chart of antenatal clinic visit count by year. See **Supplementary Table S5** for underlying data.

**
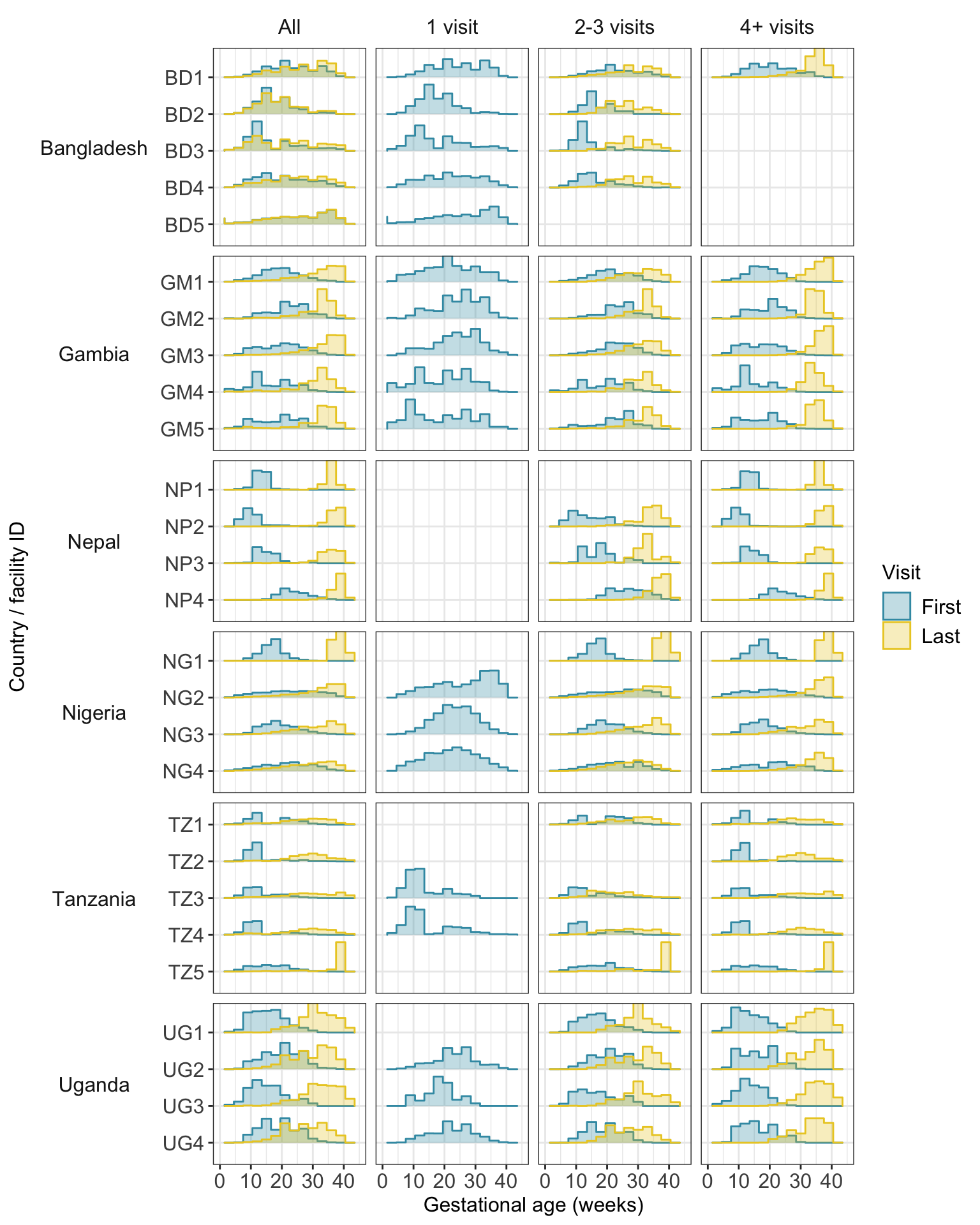
**

**Supplementary Figure S3. Distribution of gestational age at first and last antenatal clinic visits by clinic.** Density plots are shown overall and stratified by total ANC visit count (1, 2–3, 4+). Subgroups with <100 individuals are not shown. See **Supplementary Table S7** for underlying data.


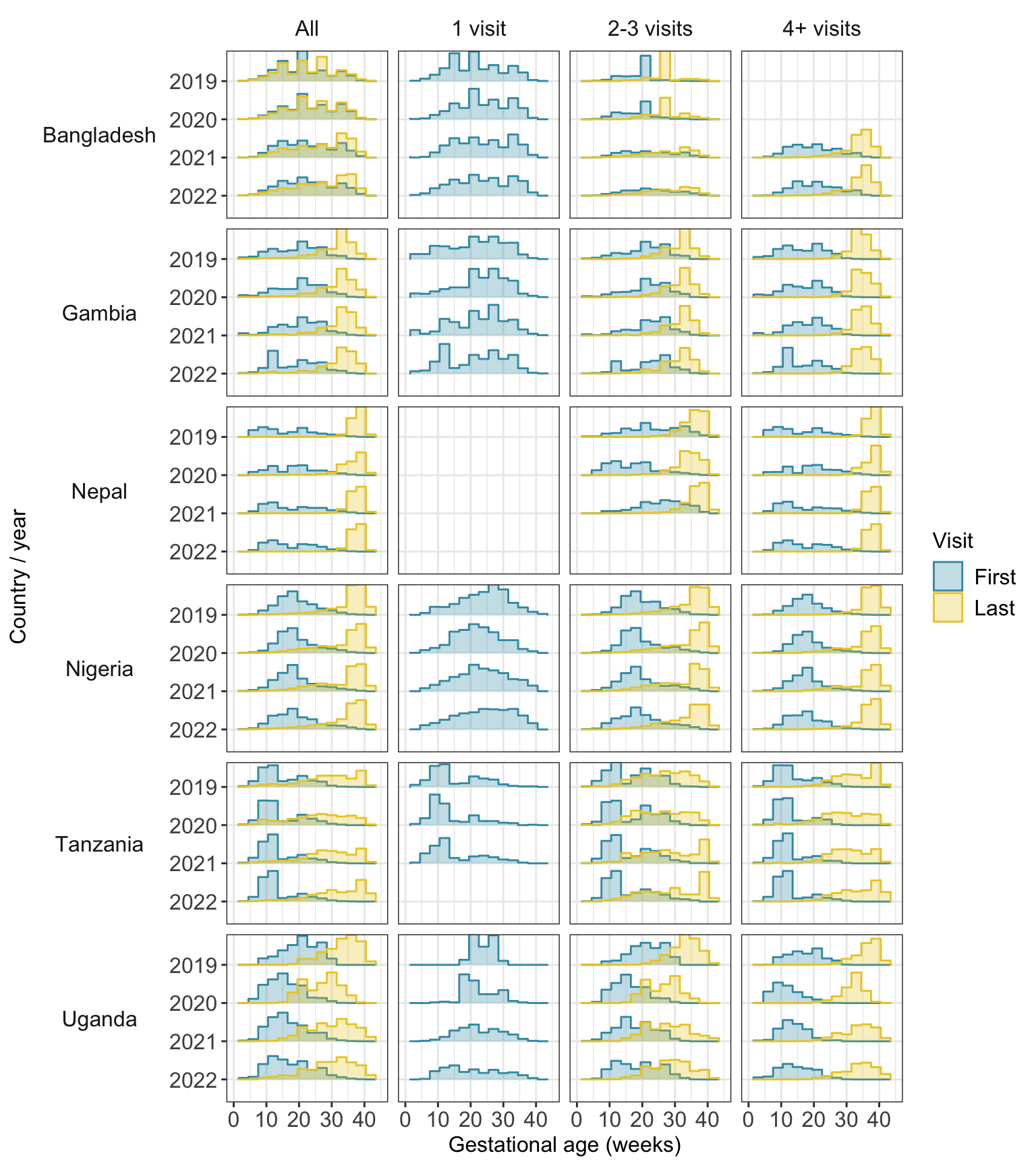


**Supplementary Figure S4. Distribution of gestational age at first and last antenatal clinic visits by year.** Density plots are shown overall and stratified by total ANC visit count (1, 2–3, 4+). Subgroups with <100 individuals are not shown. See **Supplementary Table S7** for underlying data.

**Supplementary Table S1. Interpolation approaches for unobserved visits.**

| **Approach** | **Summary** | **Example of interpolated visits by GA**  **(N visits = 4, First visit = 24 weeks, Last visit = 36 weeks)** |
| --- | --- | --- |
| Random interpolation | Unobserved visits imputed at random between first and last visit | 24, 24, 31, 36* |
| Observed data only | Only recorded dates used (i.e., GA at first and last visits) | 24, 36 |
| Even interpolation | Unobserved visits imputed at even distribution between first and last visits | 24, 28, 32, 36 |

GA, gestational age in weeks.

**Supplementary Table S2. Selection of individual-level data for inclusion in the study.**

| **Country** | **N records** | **Filter 1:**  **N visits not recorded** | **Filter 2:**  **1 visit but first GA ≠ last GA** | **Filter 3:**  **Clinic not recorded** | **N retained for analysis of ANC visit count (%)** | **Flag 4:**  **Missing first or last GA*** | **Flag 5:**  **Last GA preceded first GA** | **N retained for analysis of GA windows (%)**† |
| --- | --- | --- | --- | --- | --- | --- | --- | --- |
| Bangladesh | 34,014 | 11 | 53 | 0 | 33,950 (99.8%) | 6393 | 90 | 27,467 (80.9%) |
| Gambia | 20,930 | 0 | 11 | 1 | 20,918 (99.9%) | 87 | 70 | 20,761 (99.2%) |
| Nepal | 9645 | 0 | 0 | 0 | 9645 (100%) | 0 | 8 | 9637 (99.9%) |
| Nigeria | 42,841 | 1 | 1775 | 0 | 41,065 (95.9%) | 111 | 42 | 40,912 (99.6%) |
| Tanzania | 6336 | 0 | 4 | 0 | 6332 (99.9%) | 0 | 2 | 6330 (100%) |
| Uganda | 12,382 | 0 | 419 | 6 | 11,957 (96.6%) | 0 | 5 | 11,952 (100%) |
| Combined (sum) | 126,148 | 12 | 2262 | 7 | 123,867 (98.2%) | 6591 | 217 | 117,059 (94.5%) |

* Analysis of GA windows restricted to cases with complete data for first and last GA. † Uses N retained for analysis of ANC visit count as denominator. GA, gestational age in weeks.

**Supplementary Table S3. Antenatal clinic visit counts and gestational age determination method.**

| **Country** | **Clinic** | **N records** | **N records by year** | | | | **GA determination method** | | |
| --- | --- | --- | --- | --- | --- | --- | --- | --- | --- |
|  |  |  | **2019** | **2020** | **2021** | **2022** | **Ultrasound** | **Fundal height** | **LMP** |
| Bangladesh | BD1 | 27196 | 3064 | 2656 | 10340* | 11136* | Yes | Yes | Yes |
|  | BD2 | 2075 | 571 | 391 | 483 | 630 | Yes | Yes | Yes |
|  | BD3 | 1693 | 446 | 380 | 447 | 420 | Yes | Yes | Yes |
|  | BD4 | 1929 | 415 | 481 | 412 | 621 | Yes | Yes | Yes |
|  | BD5 | 1057 | 222 | 203 | 170 | 462 | Yes | Yes | Yes |
| Gambia | GM1 | 1367 | 485 | 571 | 1^†^ | 310 | Yes | Yes | No |
|  | GM2 | 7976 | 1627 | 1768 | 2193 | 2388 | No | Yes | No |
|  | GM3 | 2589 | 362 | 788 | 651 | 788 | Yes | Yes | Yes |
|  | GM4 | 6774 | 1724 | 1741 | 1551 | 1758 | Yes | Yes | No |
|  | GM5 | 2212 | 683 | 468 | 368 | 693 | Yes | Yes | No |
| Nepal | NP1 | 1123 | 130 | 129 | 355 | 509 | Yes | Yes | Yes |
|  | NP2 | 3202 | 962 | 658 | 651 | 931 | No | Yes | Yes |
|  | NP3 | 729 | 159 | 300 | 117 | 153 | No | Yes | Yes |
|  | NP4 | 4591 | 1196 | 859 | 1077 | 1459 | Yes | Yes | Yes |
| Nigeria | NG1 | 17390 | 4485 | 4764 | 5899 | 2242‡ | Yes | Yes | Yes |
|  | NG2 | 3712 | 41^§^ | 599^§^ | 1880 | 1192 | Yes | Yes | Yes |
|  | NG3 | 12652 | 3004 | 3154 | 3107 | 3387 | Yes | Yes | Yes |
|  | NG4 | 7311 | 2072 | 1899 | 1782 | 1558 | Yes | Yes | Yes |
| Tanzania | TZ1 | 1036 | 263 | 337 | 215 | 221 | Yes | Yes | Yes |
|  | TZ2 | 218 | 51 | 66 | 65 | 36 | Yes | Yes | Yes |
|  | TZ3 | 2019 | 474 | 415 | 455 | 675 | Yes | Yes | Yes |
|  | TZ4 | 2248 | 491 | 446 | 629 | 682 | Yes | Yes | Yes |
|  | TZ5 | 811 | 254 | 198 | 186 | 173 | Yes | Yes | Yes |
| Uganda | UG1 | 938 | 305 | 252 | 181 | 200 | No | Yes | Yes |
|  | UG2 | 2042 | 716 | 6** | 545 | 775 | Yes | Yes | Yes |
|  | UG3 | 4395 | 1004 | 1080 | 1157 | 1154 | No | Yes | Yes |
|  | UG4 | 4582 | 1073 | 1157 | 1181 | 1171 | Yes | Yes | Yes |

LMP, last menstrual period. * BD1 clinic moved to a digital system in 2021/22 which resulted in more complete data available for these years of the study; † GM1 clinic had no ANC registries available for 2021, only aggregate data were available; ‡ NG1 clinic had an increase in cost of clinic appointments in 2022 resulting in fewer attendances; § NG2 clinic opened as an ANC clinic in 2019 and was not at full capacity for the whole year; ** UG2 clinic was closed in 2020 due to Covid-19.

**Supplementary Table S4. Distribution of antenatal clinic visit counts by country.**

| **Country** | **N records** | **Median (IQR) count** | **Min. count** | **Max. count** | **N (%) with ANC visit count** | | | | | | | |
| --- | --- | --- | --- | --- | --- | --- | --- | --- | --- | --- | --- | --- |
|  |  |  |  |  | **1** | **2** | **3** | **4** | **5** | **6** | **7** | **8+** |
| Bangladesh | 33,950 | 1 (1–3) | 1 | 16* | 17,791 (52.4) | 6347 (18.7) | 4437 (13.1) | 2509 (7.4) | 1322 (3.9) | 778 (2.3) | 386 (1.1) | 380 (1.1) |
| Gambia | 20,919 | 3 (2–4) | 1 | 16* | 2975 (14.2) | 3570 (17.1) | 4798 (22.9) | 4620 (22.1) | 2907 (13.9) | 1264 (6.0) | 456 (2.2) | 329 (1.6) |
| Nepal | 9645 | 5 (4–6) | 1 | 10 | 39 (0.4) | 220 (2.3) | 922 (9.6) | 3333 (34.6) | 2707 (28.1) | 1979 (20.5) | 328 (3.4) | 117 (1.2) |
| Nigeria | 41,054 | 4 (2–6) | 1 | 14 | 4665 (11.4) | 6677 (16.3) | 6282 (15.3) | 5661 (13.8) | 4196 (10.2) | 4314 (10.5) | 2865 (7.0) | 6405 (15.6) |
| Tanzania | 6332 | 4 (3–5) | 1 | 11 | 577 (9.1) | 731 (11.5) | 930 (14.7) | 1458 (23.0) | 1142 (18.0) | 735 (11.6) | 378 (6.0) | 381 (6.0) |
| Uganda | 11,957 | 3 (2–4) | 1 | 8 | 1149 (9.6) | 3062 (25.6) | 3905 (32.7) | 2138 (17.9) | 935 (7.8) | 578 (4.8) | 153 (1.3) | 37 (0.3) |
| Combined (sum) | 123,867 | 3 (2–5) | 1 | 16* | 27,196 (22.0) | 20,607 (16.6) | 21,274 (17.2) | 19,718 (15.9) | 13,209 (10.7) | 9648 (7.8) | 4566 (3.7) | 7649 (6.2) |
| Combined (norm.) | 123,867 | 4 (2–5) | 1 | 16* | 27,196 (16.2) | 20,607 (15.2) | 21,274 (18.0) | 19,718 (19.8) | 13,209 (13.7) | 9648 (9.3) | 4566 (3.5) | 7649 (4.3) |

* Individuals with >16 visits were excluded from the database. ANC, antenatal clinic; IQR, interquartile range; Max., Maximum; Min., Minimum; Norm., Normalised by inverse probability weighted scaling at country level.

**Supplementary Table S5. Distribution of antenatal clinic visit counts by clinic.**

| **Country** | **Clinic** | **N records** | **Median (IQR) count** | **Min. count** | **Max. count** | **N (%) with ANC visit count** | | | | | | | |
| --- | --- | --- | --- | --- | --- | --- | --- | --- | --- | --- | --- | --- | --- |
|  |  |  |  |  |  | **1** | **2** | **3** | **4** | **5** | **6** | **7** | **8+** |
| Bangladesh | BD1 | 27196 | 2 (1-3) | 1 | 15 | 13020 (47.9) | 5287 (19.4) | 3911 (14.4) | 2125 (7.8) | 1314 (4.8) | 777 (2.9) | 386 (1.4) | 376 (1.4) |
|  | BD2 | 2075 | 1 (1-1) | 1 | 5 | 1734 (83.6) | 197 (9.5) | 93 (4.5) | 43 (2.1) | 8 (0.4) | 0 (0) | 0 (0) | 0 (0) |
|  | BD3 | 1693 | 1 (1-2) | 1 | 8 | 890 (52.6) | 410 (24.2) | 230 (13.6) | 161 (9.5) | 0 (0) | 1 (0.1) | 0 (0) | 1 (0.1) |
|  | BD4 | 1929 | 1 (1-2) | 1 | 16* | 1126 (58.4) | 425 (22) | 197 (10.2) | 178 (9.2) | 0 (0) | 0 (0) | 0 (0) | 3 (0.2) |
|  | BD5 | 1057 | 1 (1-1) | 1 | 4 | 1021 (96.6) | 28 (2.6) | 6 (0.6) | 2 (0.2) | 0 (0) | 0 (0) | 0 (0) | 0 (0) |
| Gambia | GM1 | 1367 | 4 (2-5) | 1 | 9 | 231 (16.9) | 184 (13.5) | 256 (18.7) | 295 (21.6) | 191 (14) | 103 (7.5) | 67 (4.9) | 40 (2.9) |
|  | GM2 | 7976 | 3 (2-4) | 1 | 8 | 1234 (15.5) | 1675 (21) | 2205 (27.6) | 1821 (22.8) | 816 (10.2) | 183 (2.3) | 40 (0.5) | 2 (0) |
|  | GM3 | 2589 | 4 (3-6) | 1 | 8 | 326 (12.6) | 290 (11.2) | 428 (16.5) | 425 (16.4) | 355 (13.7) | 297 (11.5) | 213 (8.2) | 255 (9.8) |
|  | GM4 | 6774 | 3 (2-4) | 1 | 16* | 900 (13.3) | 1141 (16.8) | 1526 (22.5) | 1597 (23.6) | 1082 (16) | 453 (6.7) | 65 (1) | 10 (0.1) |
|  | GM5 | 2212 | 4 (2-5) | 1 | 8 | 284 (12.8) | 280 (12.7) | 383 (17.3) | 481 (21.7) | 463 (20.9) | 228 (10.3) | 71 (3.2) | 22 (1) |
| Nepal | NP1 | 1123 | 4 (4-4) | 1 | 10 | 4 (0.4) | 6 (0.5) | 58 (5.2) | 869 (77.4) | 77 (6.9) | 57 (5.1) | 32 (2.8) | 20 (1.8) |
|  | NP2 | 3202 | 5 (5-6) | 1 | 6 | 21 (0.7) | 137 (4.3) | 319 (10) | 307 (9.6) | 1257 (39.3) | 1161 (36.3) | 0 (0) | 0 (0) |
|  | NP3 | 729 | 4 (4-4) | 1 | 6 | 13 (1.8) | 40 (5.5) | 94 (12.9) | 512 (70.2) | 57 (7.8) | 13 (1.8) | 0 (0) | 0 (0) |
|  | NP4 | 4591 | 5 (4-5) | 1 | 9 | 1 (0) | 37 (0.8) | 451 (9.8) | 1645 (35.8) | 1316 (28.7) | 748 (16.3) | 296 (6.4) | 97 (2.1) |
| Nigeria | NG1 | 17390 | 5 (3-7) | 2 | 12 | 0 (0) | 2762 (15.9) | 2510 (14.4) | 2773 (15.9) | 1709 (9.8) | 2180 (12.5) | 1646 (9.5) | 3810 (21.9) |
|  | NG2 | 3712 | 3 (2-5) | 1 | 12 | 682 (18.4) | 785 (21.1) | 740 (19.9) | 504 (13.6) | 274 (7.4) | 201 (5.4) | 193 (5.2) | 333 (9) |
|  | NG3 | 12652 | 4 (2-6) | 1 | 12 | 1858 (14.7) | 1655 (13.1) | 1948 (15.4) | 1600 (12.6) | 1654 (13.1) | 1510 (11.9) | 722 (5.7) | 1705 (13.5) |
|  | NG4 | 7311 | 3 (1-5) | 1 | 14 | 2125 (29.1) | 1475 (20.2) | 1084 (14.8) | 784 (10.7) | 559 (7.6) | 423 (5.8) | 304 (4.2) | 557 (7.6) |
| Tanzania | TZ1 | 1036 | 3 (2-4) | 1 | 8 | 85 (8.2) | 184 (17.8) | 285 (27.5) | 303 (29.2) | 135 (13) | 33 (3.2) | 10 (1) | 1 (0.1) |
|  | TZ2 | 218 | 4 (3-5) | 1 | 8 | 10 (4.6) | 14 (6.4) | 32 (14.7) | 58 (26.6) | 66 (30.3) | 22 (10.1) | 14 (6.4) | 2 (0.9) |
|  | TZ3 | 2019 | 5 (3-6) | 1 | 11 | 182 (9) | 156 (7.7) | 206 (10.2) | 422 (20.9) | 363 (18) | 251 (12.4) | 132 (6.5) | 307 (15.2) |
|  | TZ4 | 2248 | 4 (3-5) | 1 | 10 | 266 (11.8) | 210 (9.3) | 322 (14.3) | 569 (25.3) | 459 (20.4) | 276 (12.3) | 112 (5) | 34 (1.5) |
|  | TZ5 | 811 | 5 (3-6) | 1 | 9 | 34 (4.2) | 167 (20.6) | 85 (10.5) | 106 (13.1) | 119 (14.7) | 153 (18.9) | 110 (13.6) | 37 (4.6) |
| Uganda | UG1 | 938 | 3 (3-4) | 1 | 8 | 21 (2.2) | 137 (14.6) | 402 (42.9) | 215 (22.9) | 95 (10.1) | 41 (4.4) | 22 (2.3) | 5 (0.5) |
|  | UG2 | 2042 | 3 (2-3) | 1 | 6 | 296 (14.5) | 673 (33) | 639 (31.3) | 303 (14.8) | 112 (5.5) | 19 (0.9) | 0 (0) | 0 (0) |
|  | UG3 | 4395 | 3 (3-5) | 1 | 8 | 112 (2.5) | 728 (16.6) | 1419 (32.3) | 953 (21.7) | 539 (12.3) | 488 (11.1) | 128 (2.9) | 28 (0.6) |
|  | UG4 | 4582 | 3 (2-3) | 1 | 8 | 720 (15.7) | 1524 (33.3) | 1445 (31.5) | 667 (14.6) | 189 (4.1) | 30 (0.7) | 3 (0.1) | 4 (0.1) |

* Individuals with >16 visits were excluded from the database. ANC, antenatal clinic; IQR, interquartile range; Max., Maximum; Min., Minimum.

**Supplementary Table S6. Distribution of antenatal clinic visit counts by year.**

| **Country** |  | **N records** | **Median (IQR) count** | **Min. count** | **Max. count** | **N (%) with ANC visit count** | | | | | | | |
| --- | --- | --- | --- | --- | --- | --- | --- | --- | --- | --- | --- | --- | --- |
|  | **Year** |  |  |  |  | **1** | **2** | **3** | **4** | **5** | **6** | **7** | **8+** |
| Bangladesh | 2019 | 4718 | 1 (1-1) | 1 | 6 | 3805 (80.6) | 257 (5.4) | 597 (12.7) | 56 (1.2) | 2 (0) | 1 (0) | 0 (0) | 0 (0) |
|  | 2020 | 4111 | 1 (1-1) | 1 | 5 | 3654 (88.9) | 204 (5) | 178 (4.3) | 72 (1.8) | 3 (0.1) | 0 (0) | 0 (0) | 0 (0) |
|  | 2021 | 11852 | 2 (1-3) | 1 | 12 | 4742 (40) | 2817 (23.8) | 1803 (15.2) | 1128 (9.5) | 622 (5.2) | 366 (3.1) | 191 (1.6) | 183 (1.5) |
|  | 2022 | 13269 | 2 (1-3) | 1 | 16* | 5590 (42.1) | 3069 (23.1) | 1859 (14) | 1253 (9.4) | 695 (5.2) | 411 (3.1) | 195 (1.5) | 197 (1.5) |
| Gambia | 2019 | 4881 | 3 (2-4) | 1 | 8 | 778 (15.9) | 890 (18.2) | 1305 (26.7) | 1180 (24.2) | 524 (10.7) | 175 (3.6) | 27 (0.6) | 2 (0) |
|  | 2020 | 5336 | 3 (2-4) | 1 | 8 | 916 (17.2) | 1060 (19.9) | 1336 (25) | 1094 (20.5) | 616 (11.5) | 235 (4.4) | 62 (1.2) | 17 (0.3) |
|  | 2021 | 4764 | 3 (2-5) | 1 | 15 | 565 (11.9) | 816 (17.1) | 1056 (22.2) | 1071 (22.5) | 778 (16.3) | 321 (6.7) | 89 (1.9) | 68 (1.4) |
|  | 2022 | 5937 | 4 (2-5) | 1 | 16* | 716 (12.1) | 804 (13.5) | 1101 (18.5) | 1274 (21.5) | 989 (16.7) | 533 (9) | 278 (4.7) | 242 (4.1) |
| Nepal | 2019 | 2447 | 5 (4-6) | 1 | 10 | 2 (0.1) | 14 (0.6) | 125 (5.1) | 565 (23.1) | 836 (34.2) | 748 (30.6) | 117 (4.8) | 40 (1.6) |
|  | 2020 | 1946 | 4 (3-4) | 1 | 9 | 33 (1.7) | 189 (9.7) | 508 (26.1) | 749 (38.5) | 257 (13.2) | 136 (7) | 56 (2.9) | 18 (0.9) |
|  | 2021 | 2200 | 4 (4-5) | 1 | 8 | 2 (0.1) | 16 (0.7) | 192 (8.7) | 921 (41.9) | 680 (30.9) | 348 (15.8) | 35 (1.6) | 6 (0.3) |
|  | 2022 | 3052 | 5 (4-6) | 1 | 9 | 2 (0.1) | 1 (0) | 97 (3.2) | 1098 (36) | 934 (30.6) | 747 (24.5) | 120 (3.9) | 53 (1.7) |
| Nigeria | 2019 | 9602 | 4 (2-6) | 1 | 14 | 915 (9.5) | 1670 (17.4) | 1575 (16.4) | 1314 (13.7) | 869 (9.1) | 924 (9.6) | 613 (6.4) | 1722 (17.9) |
|  | 2020 | 10416 | 4 (2-6) | 1 | 13 | 1247 (12) | 1685 (16.2) | 1530 (14.7) | 1493 (14.3) | 986 (9.5) | 1189 (11.4) | 669 (6.4) | 1617 (15.5) |
|  | 2021 | 12668 | 4 (2-6) | 1 | 13 | 1340 (10.6) | 1899 (15) | 1791 (14.1) | 1668 (13.2) | 1441 (11.4) | 1462 (11.5) | 1121 (8.8) | 1946 (15.4) |
|  | 2022 | 8379 | 4 (2-6) | 1 | 13 | 1163 (13.9) | 1423 (17) | 1386 (16.5) | 1186 (14.2) | 900 (10.7) | 739 (8.8) | 462 (5.5) | 1120 (13.4) |
| Tanzania | 2019 | 1533 | 4 (3-5) | 1 | 9 | 172 (11.2) | 180 (11.7) | 283 (18.5) | 435 (28.4) | 295 (19.2) | 110 (7.2) | 44 (2.9) | 14 (0.9) |
|  | 2020 | 1462 | 4 (2-5) | 1 | 8 | 222 (15.2) | 200 (13.7) | 268 (18.3) | 403 (27.6) | 211 (14.4) | 103 (7) | 48 (3.3) | 7 (0.5) |
|  | 2021 | 1550 | 4 (3-6) | 1 | 10 | 118 (7.6) | 193 (12.5) | 173 (11.2) | 304 (19.6) | 321 (20.7) | 279 (18) | 111 (7.2) | 51 (3.3) |
|  | 2022 | 1787 | 5 (4-7) | 1 | 11 | 65 (3.6) | 158 (8.8) | 206 (11.5) | 316 (17.7) | 315 (17.6) | 243 (13.6) | 175 (9.8) | 309 (17.3) |
| Uganda | 2019 | 3098 | 3 (2-4) | 1 | 6 | 346 (11.2) | 635 (20.5) | 1198 (38.7) | 711 (23) | 183 (5.9) | 25 (0.8) | 0 (0) | 0 (0) |
|  | 2020 | 2495 | 3 (2-3) | 1 | 6 | 128 (5.1) | 1087 (43.6) | 888 (35.6) | 264 (10.6) | 107 (4.3) | 21 (0.8) | 0 (0) | 0 (0) |
|  | 2021 | 3064 | 3 (2-5) | 1 | 8 | 220 (7.2) | 729 (23.8) | 857 (28) | 477 (15.6) | 351 (11.5) | 346 (11.3) | 71 (2.3) | 13 (0.4) |
|  | 2022 | 3300 | 3 (2-4) | 1 | 8 | 455 (13.8) | 611 (18.5) | 962 (29.2) | 686 (20.8) | 294 (8.9) | 186 (5.6) | 82 (2.5) | 24 (0.7) |

* Individuals with >16 visits were excluded from the database. ANC, antenatal clinic; IQR, interquartile range; Max., Maximum; Min., Minimum.

**Supplementary Table S7. Distribution of gestational age at first and last antenatal clinic visit by country.**

| **Country** | **All individuals** | | | **Individuals with 1 ANC visit** | | **Individuals with 2–3 ANC visits** | | | **Individuals with 4+ ANC visits** | | | |
| --- | --- | --- | --- | --- | --- | --- | --- | --- | --- | --- | --- | --- |
|  | **N records** | **GA first, median (IQR)** | **GA last, median (IQR)** | **N records** | **GA, median (IQR)** | **N records** | **GA first, median (IQR)** | **GA last, median (IQR)** | **N records** | **GA first, median (IQR)** | **GA last, median (IQR)** |  |
| Bangladesh | 27,467 | 22 (16–28) | 27 (20–34) | 16,507 | 22 (16–30) | 7152 | 20 (16–27) | 28 (24–34) | 3808 | 20 (14–25) | 36 (32–36) |  |
| Gambia | 20,761 | 20 (14–24) | 34 (30–36) | 2890 | 24 (16–28) | 8302 | 23 (18–26) | 32 (28–34) | 9569 | 18 (12–22) | 35 (33–36) |  |
| Nepal | 9637 | 17 (11–23) | 38 (36–39) | 39 | 21 (17–26) | 1140 | 20 (13–26) | 36 (33–38) | 8458 | 16 (11–23) | 38 (36–39) |  |
| Nigeria | 40,912 | 18 (15–22) | 36 (30–38) | 4592 | 24 (18–29) | 12,918 | 19 (16–24) | 36 (30–38) | 23,402 | 17 (15–20) | 37 (35–39) |  |
| Tanzania | 6330 | 11 (10–19) | 30 (24–37) | 577 | 11 (10–20) | 1661 | 14 (11–22) | 28 (20–34) | 4092 | 11 (10–16) | 32 (27–38) |  |
| Uganda | 11,952 | 17 (12–22) | 30 (26–36) | 1,149 | 22 (18–26) | 6,962 | 18 (14–24) | 30 (24–34) | 3841 | 14 (11–18) | 35 (31–38) |  |
| Combined (sum) | 117,059 | 19 (14–24) | 34 (26–37) | 25,754 | 23 (16–30) | 38,135 | 20 (16–25) | 32 (27–36) | 53,170 | 17 (12–21) | 36 (34–38) |  |
| Combined (norm.) | 117,059 | 18 (12–24) | 34 (27–37) | 25,754 | 22 (16–29) | 38,135 | 20 (14–24) | 31 (26–36) | 53,170 | 16 (11–21) | 36 (33–38) |  |

Results are shown overall and stratified by total ANC visit count (1, 2–3, 4+). ANC, antenatal clinic; GA, gestational age in weeks; IQR, interquartile range; Norm., Normalised by inverse probability weighted scaling at country level.

**Supplementary Table S8. Distribution of gestational age at first and last antenatal clinic visit by clinic.**

| **Country** |  | **All individuals** | | | **Individuals with 1 ANC visit** | | **Individuals with 2–3 ANC visits** | | | **Individuals with 4+ ANC visits** | | | |
| --- | --- | --- | --- | --- | --- | --- | --- | --- | --- | --- | --- | --- | --- |
|  | **Clinic** | **N records** | **GA first, median (IQR)** | **GA last, median (IQR)** | **N records** | **GA, median (IQR)** | **N records** | **GA first, median (IQR)** | **GA last, median (IQR)** | **N records** | **GA first, median (IQR)** | **GA last, median (IQR)** |  |
| Bangladesh | BD1 | 21899 | 22 (18–30) | 28 (21–34) | 12099 | 24 (18–32) | 6209 | 22 (18–28) | 28 (24–34) | 3591 | 20 (15–25) | 35 (32–36) |  |
|  | BD2 | 2045 | 16 (14–20) | 18 (14–22) | 1730 | 17 (14–20) | 268 | 16 (14–20) | 26 (20–30) | 47 | – | – |  |
|  | BD3 | 1214 | 12 (12–20) | 20 (12–29) | 858 | 16 (12–24) | 287 | 12 (12–14) | 28 (24–34) | 69 | – | – |  |
|  | BD4 | 1556 | 20 (14–27) | 25 (18–32) | 1097 | 22 (16–29) | 360 | 14 (12–20) | 28 (23.5–33) | 99 | – | – |  |
|  | BD5 | 753 | 26 (17–34) | 27 (18–34) | 723 | 27 (18–34) | 28 | – | – | 2 | – | – |  |
| Gambia | GM1 | 1362 | 19 (15–24) | 34 (28–37) | 226 | 22 (15–28) | 440 | 21 (17–26) | 32 (26–36) | 696 | 18 (14–22) | 36 (33–38) |  |
|  | GM2 | 7837 | 23 (19–26) | 34 (30–36) | 1156 | 26 (20–30) | 3824 | 24 (20–28) | 32 (30–34) | 2857 | 20 (16–24) | 34 (34–36) |  |
|  | GM3 | 2586 | 20 (14–25) | 35 (31–38) | 326 | 25 (20–30) | 715 | 24 (20–27) | 32 (28–35) | 1545 | 17 (12–22) | 37 (35–38) |  |
|  | GM4 | 6770 | 16 (12–22) | 32 (28–34) | 899 | 20 (12–26) | 2664 | 20 (12–24) | 30 (28–34) | 3207 | 12 (12–19) | 34 (32–36) |  |
|  | GM5 | 2206 | 20 (12–24) | 34 (30–36) | 283 | 18 (10–28) | 659 | 24 (19–28) | 32 (29–36) | 1264 | 18 (12–22) | 36 (34–36) |  |
| Nepal | NP1 | 1123 | 13 (13–16) | 36 (35–36) | 4 | – | 64 | – | – | 1055 | 13 (13–16) | 36 (36–36) |  |
|  | NP2 | 3201 | 10 (8–12) | 37 (36–39) | 21 | – | 455 | 13 (9–19) | 34 (32–37) | 2725 | 9 (8–11) | 38 (37–39) |  |
|  | NP3 | 729 | 14 (13–17) | 36 (34–38) | 13 | – | 134 | 17 (13–21) | 34 (30–34) | 582 | 14 (13–16) | 36 (35–38) |  |
|  | NP4 | 4584 | 23 (21–27) | 38 (37–40) | 1 | – | 487 | 27 (22–31) | 37 (36–39) | 4096 | 23 (21–27) | 39 (37–40) |  |
| Nigeria | NG1 | 17375 | 17 (15–19) | 38 (37–40) | 0 | – | 5266 | 17 (15–19) | 38 (37–40) | 12109 | 17 (15–19) | 38 (37–40) |  |
|  | NG2 | 3703 | 23 (15–30) | 34 (28–37) | 682 | 29 (19–34) | 1520 | 25 (17–31) | 32 (26–36) | 1501 | 19 (13–25) | 36 (33–38) |  |
|  | NG3 | 12641 | 18 (15–23) | 33 (26–37) | 1858 | 24 (19–28) | 3600 | 19 (16–24) | 33 (28–37) | 7183 | 18 (14–21) | 34 (28–37) |  |
|  | NG4 | 7193 | 22 (16–28) | 30 (23–35) | 2052 | 23 (16–28) | 2532 | 22 (17–29) | 28 (23–32) | 2609 | 21 (15–28) | 35 (32–37) |  |
| Tanzania | TZ1 | 1035 | 12 (12–23) | 28 (22–33) | 85 | – | 469 | 20 (12–24) | 29 (22–33) | 481 | 12 (11–20) | 29 (25–34) |  |
|  | TZ2 | 218 | 11 (10–11) | 29 (25–33) | 10 | – | 46 | – | – | 162 | 11 (10–11) | 29 (28–33) |  |
|  | TZ3 | 2018 | 11 (10–18) | 29 (23–37) | 182 | 11 (9–16) | 362 | 11 (10–19) | 21 (16–27) | 1474 | 11 (10–17) | 32 (27–38) |  |
|  | TZ4 | 2248 | 11 (10–11) | 29 (24–34) | 266 | 11 (10–21) | 532 | 11 (10–21) | 27 (21–33) | 1450 | 11 (10–11) | 31 (27–35) |  |
|  | TZ5 | 811 | 16 (11–20) | 39 (38–39) | 34 | – | 252 | 18 (13–22) | 38 (34.5–39) | 525 | 16 (11–20) | 39 (38–39) |  |
| Uganda | UG1 | 938 | 16 (11–19) | 31 (28–36) | 21 | – | 539 | 17 (12–20) | 30 (27–33) | 378 | 13 (10–18) | 35 (31–38) |  |
|  | UG2 | 2039 | 20 (16–24) | 30 (24–36) | 296 | 24 (20–28) | 1309 | 20 (16–24) | 31 (24–34) | 434 | 16 (12–20) | 34 (29–37) |  |
|  | UG3 | 4395 | 15 (11–19) | 32 (29–37) | 112 | 19 (16–21) | 2147 | 16 (11–22) | 30 (27–35) | 2136 | 14 (11–17) | 35 (32–38) |  |
|  | UG4 | 4580 | 18 (14–24) | 28 (22–34) | 720 | 22 (18–27) | 2967 | 18 (14–24) | 28 (22–32) | 893 | 16 (12–20) | 34 (30–37) |  |

Results are shown overall and stratified by total ANC visit count (1, 2–3, 4+). Metrics for subgroups with <100 individuals are not shown. ANC, antenatal clinic; GA, gestational age in weeks; IQR, interquartile range; Norm., Normalised by inverse probability weighted scaling at country level.

**Supplementary Table S13. Proportion of individuals with at least one antenatal clinic visit in specific gestational age windows by total antenatal clinic visit count.**

| **GA window** | **Country** | **1–3 ANC visits** | | **4+ ANC visits** | | **% gain**  **(4+ vs 1–3 ANC visits)** |
| --- | --- | --- | --- | --- | --- | --- |
|  |  | **N** | **n (%)** | **N** | **n (%)** |  |
| **0–23 weeks** | Bangladesh | 23659 | 12901 (54.5) | 3808 | 2619 (68.8) | 14.3 |
|  | Gambia | 11192 | 5669 (50.7) | 9569 | 7787 (81.4) | 30.7 |
|  | Nepal | 1179 | 792 (67.2) | 8458 | 6585 (77.9) | 10.7 |
|  | Nigeria | 17510 | 11945 (68.2) | 23402 | 20097 (85.9) | 17.7 |
|  | Tanzania | 2238 | 1793 (80.1) | 4092 | 3829 (93.6) | 13.5 |
|  | Uganda | 8111 | 5817 (71.7) | 3841 | 3608 (93.9) | 22.2 |
|  | Combined (sum) | 63889 | 38917 (60.9) | 53170 | 44525 (83.7) | 22.8 |
|  | Combined (norm.) | 63889 | – (65.4) | 53170 | – (83.6) | 18.2 |
| **24–36 weeks** | Bangladesh | 23659 | 12884 (54.5) | 3808 | 3646 (95.7) | 41.2 |
|  | Gambia | 11192 | 9170 (81.9) | 9569 | 9446 (98.7) | 16.8 |
|  | Nepal | 1179 | 1028 (87.2) | 8458 | 8002 (94.6) | 7.4 |
|  | Nigeria | 17510 | 10151 (58) | 23402 | 21789 (93.1) | 35.1 |
|  | Tanzania | 2238 | 1061 (47.4) | 4092 | 3598 (87.9) | 40.5 |
|  | Uganda | 8111 | 5891 (72.6) | 3841 | 3629 (94.5) | 21.9 |
|  | Combined (sum) | 63889 | 40185 (62.9) | 53170 | 50110 (94.2) | 31.3 |
|  | Combined (norm.) | 63889 | – (66.9) | 53170 | – (94.1) | 27.2 |
| **28–36 weeks** | Bangladesh | 23659 | 9412 (39.8) | 3808 | 3359 (88.2) | 48.4 |
|  | Gambia | 11192 | 7555 (67.5) | 9569 | 9067 (94.8) | 27.3 |
|  | Nepal | 1179 | 909 (77.1) | 8458 | 7379 (87.2) | 10.1 |
|  | Nigeria | 17510 | 7645 (43.7) | 23402 | 19445 (83.1) | 39.4 |
|  | Tanzania | 2238 | 714 (31.9) | 4092 | 2839 (69.4) | 37.5 |
|  | Uganda | 8111 | 4498 (55.5) | 3841 | 3185 (82.9) | 27.4 |
|  | Combined (sum) | 63889 | 30733 (48.1) | 53170 | 45274 (85.1) | 37.0 |
|  | Combined (norm.) | 63889 | – (52.6) | 53170 | – (84.3) | 31.7 |
| **32–36 weeks** | Bangladesh | 23659 | 5803 (24.5) | 3808 | 2769 (72.7) | 48.2 |
|  | Gambia | 11192 | 5004 (44.7) | 9569 | 7484 (78.2) | 33.5 |
|  | Nepal | 1179 | 702 (59.5) | 8458 | 5993 (70.9) | 11.4 |
|  | Nigeria | 17510 | 4791 (27.4) | 23402 | 15150 (64.7) | 37.3 |
|  | Tanzania | 2238 | 367 (16.4) | 4092 | 1674 (40.9) | 24.5 |
|  | Uganda | 8111 | 2137 (26.3) | 3841 | 2240 (58.3) | 32.0 |
|  | Combined (sum) | 63889 | 18804 (29.4) | 53170 | 35310 (66.4) | 37.0 |
|  | Combined (norm.) | 63889 | – (33.1) | 53170 | – (64.3) | 31.2 |

ANC, antenatal clinic; GA, gestational age in weeks; Norm., normalised by taking mean of country-specific estimates.
